# Down-Regulation of Surface CD28 under Belatacept Treatment: An Escape Mechanism for Antigen-Reactive T-Cells

**DOI:** 10.1371/journal.pone.0148604

**Published:** 2016-02-26

**Authors:** Gretchen N. de Graav, Dennis A. Hesselink, Marjolein Dieterich, Rens Kraaijeveld, Willem Weimar, Carla C. Baan

**Affiliations:** Department of Internal Medicine, Section of Transplantation and Nephrology, Erasmus MC, University Medical Center, Rotterdam, the Netherlands; Beth Israel Deaconess Medical Center, Harvard Medical School, UNITED STATES

## Abstract

**Background:**

The co-stimulatory inhibitor of the CD28-CD80/86-pathway, belatacept, allows calcineurin-inhibitor-free immunosuppression in kidney transplantation. However, aggressive T-cell mediated allogeneic responses have been observed in belatacept-treated patients, which could be explained by effector-memory T-cells that lack membrane expression of CD28, *i*.*e*. CD28-negative (CD28^NULL^) T-cells. CD28-positive (CD28^POS^) T-cells that down regulate their surface CD28 after allogeneic stimulation could also pose a threat against the renal graft. The aim of this study was to investigate this potential escape mechanism for CD28^POS^ T-cells under belatacept treatment.

**Materials & Methods:**

PBMCs, isolated T-cell memory subsets and isolated CD28^POS^ T-cells were obtained from end-stage renal disease (ESRD) patients and co-cultured with allo-antigen in the presence of belatacept to mimic allogeneic reactions in kidney-transplant patients under belatacept treatment. As a control, IgG was used in the absence of belatacept.

**Results:**

Despite high *in vitro* belatacept concentrations, a residual T-cell growth of ±30% was observed compared to the IgG control after allogeneic stimulation. Of the alloreactive T-cells, the majority expressed an effector-memory phenotype. This predominance for effector-memory T-cells within the proliferated cells was even larger when a higher dose of belatacept was added. Contrary to isolated naïve and central-memory T cells, isolated effector-memory T cells could not be inhibited by belatacept in differentiation or allogeneic IFNγ production. The proportion of CD28-positive T cells was lower within the proliferated T cell population, but was still substantial. A fair number of the isolated initially CD28^POS^ T-cells differentiated into CD28^NULL^ T-cells, which made them not targetable by belatacept. These induced CD28^NULL^ T-cells were not anergic as they produced high amounts of IFNγ upon allogeneic stimulation. The majority of the proliferated isolated originally CD28^POS^ T-cells, however, still expressed CD28 and also expressed IFNγ.

**Conclusion:**

This study provides evidence that, apart from CD28^NULL^ T-cells, also CD28^POS^, mostly effector-memory T-cells can mediate allogeneic responses despite belatacept treatment.

## Introduction

The co-stimulatory inhibitor of the CD28-CD80/86-pathway, belatacept, is a promising alternative for calcineurin-inhibitors in kidney transplantation.[1–3] This co-stimulatory inhibitor does not directly down-regulate or block CD28 on T-cells, but induces T-cell anergy by depriving T-cells from the necessary co-stimulatory signal from CD80/86 on antigen-presenting cells.[4] Aggressive T cell-mediated allogeneic responses have been observed in belatacept-treated patients.[1] This phenomenon can be explained by the actions of memory T-cells that are less or not susceptible to co-stimulatory blockade of the CD28-CD80/86 pathway.[5, 6] *In vitro* studies demonstrated that, despite the presence of belatacept, effector-memory T-cells which lack membrane expression of CD28, *i*.*e*. CD28-negative (CD28^NULL^) T-cells, produce high levels of effector cytokines upon allogeneic stimulation.[6–8] CD28^POS^ T-cells can down regulate their surface CD28 when the transcriptional initiator element of CD28 is disrupted [9], which occurs after repeated antigen-stimulation (*e*.*g*. as the result of physiological aging, chronic viral infection, malignancy, auto-immunity, and transplantation).[10] So, in belatacept-treated patients, in addition to pre-existing CD28^NULL^ T-cells, CD28^POS^ T-cells that down regulate their surface CD28 after allogeneic stimulation could also pose a threat to the renal graft. In solid organ transplantation, seemingly opposing functions of CD28^NULL^ T-cells have been reported. These cells can have immunoregulatory functions, [11, 12], show features of immunoquiescence [10], as well as mediate allogeneic or anti-viral immune responses.[5–7, 13, 14] One study reported CD4^POS^CD28^NULL^ T-cells could play an important role in glucocorticoid-resistant rejection occurring during belatacept treatment.[8] No studies on peripheral blood mononuclear cells (PBMCs) derived from end-stage renal disease (ESRD) patients have been conducted to determine the ability of their CD28^POS^ T-cells to down regulate surface CD28 in the presence of belatacept, making them resistant to blockade of the CD28-CD80/86-pathway.

The aim of this study was to investigate a potential escape mechanism for CD28^POS^ T-cells under belatacept treatment, *i*.*e*. the down regulation of surface CD28 by these cells after allogeneic stimulation. PBMCs, isolated T-cell memory subsets and isolated CD28^POS^ T-cells were obtained from ESRD patients (one day before kidney-transplantation) and co-cultured with donor antigen in the presence or absence of belatacept to mimic allogeneic reactions in kidney transplant patients under belatacept treatment, and therefore explain the aggressive T cell-mediated responses in these patients.[1]

## Materials and Methods

### Study population and materials

Defrosted PBMCs from patients sampled one day before kidney transplantation were analyzed. This study was approved by the Medical Ethical Committee of the Erasmus MC University Medical Center in Rotterdam, the Netherlands (MEC-2007-228, MEC-2010-022). All patients gave written consent to collect their biomaterial as part of the ongoing transplant biobank programs. None of the transplant donors were from a vulnerable population and all donors or next of kin provided written informed consent that was freely given. Samples were randomly selected when enough patient and donor material were available, and when patient and donor were not identical for HLA class II. The patient characteristics are depicted in Table A in S1 File. Materials of 61 patients were used for the PBMC study (*n* = 33), for the isolated T-cell memory subset study (*n* = 4) and for the isolated CD28^POS^ T-cell study (*n* = 24).

### Flow cytometric isolation of recipients’ PBMCs

By use of an AriaII FACS sorter^™^ (Becton Dickinson, BD, Franklin Lakes, NJ), pure CD28^POS^ cells (purity 98% [95–100%]) were isolated. PBMCs were stained with CD3 Brilliant Violet 510 (BioLegend, San Diego, CA), CD4 Pacific Blue (BD, Franklin Lakes, NJ), CD8 APC-Cy7 (BD Pharm, San Diego, CA), CD28 APC (BD), and the viability dye 7-AAD PerCP (BD). Pure memory subsets (≥95% pure) were isolated using CD3 Brilliant Violet 510 (BioLegend), CD45RO PE-Cy7 (BD) and CCR7 PE (BD): naïve (T_N_ cells: CCR7+CD45RO-), central-memory (T_CM_ cells: CCR7+, CD45RO+), effector-memory (T_EM_ cells: CCR7-, CD45RO+), and end-stage terminally-differentiated EMRA (T_EMRA_ cells: CCR7-CD45RO-) T-cells.

### Mixed lymphocyte reactions (MLRs)

**Fig 1:**
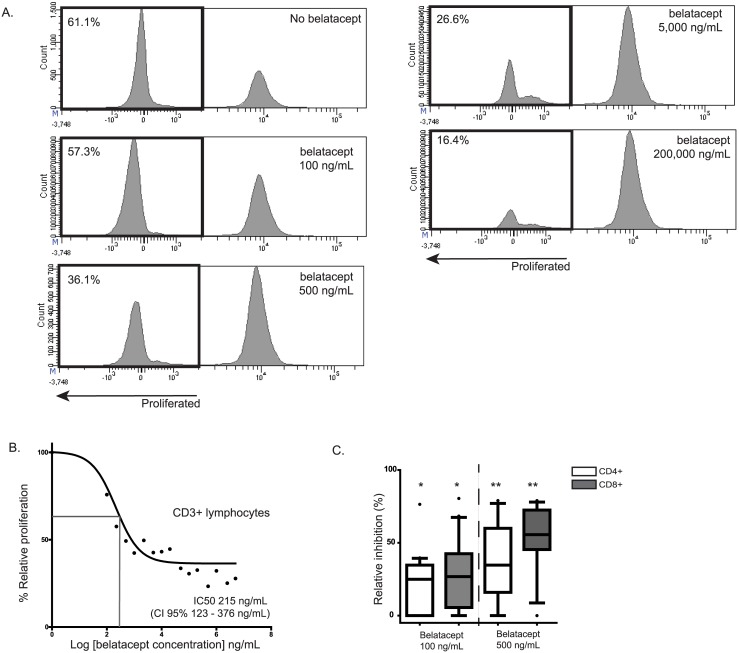
Despite the dose-dependent inhibition by belatacept of T-cell proliferation, residual T-cell proliferation is present despite high doses of belatacept. Experiments were performed with PBMCs derived from healthy volunteers (n = 6). A representative sample is shown (A). The IC_50_ was calculated using a logarithmic transformation of belatacept concentrations (log [inhibitor]- *versus*- response curve) (B). The relative inhibition by belatacept in MLRs with patients’ PBMCs is given for CD4^POS^ and CD8^POS^ T-cells. The human IgG control has been set at the zero-line (C). N.B.: In (C) black lines represent the medians. The upper and lower borders of the boxes represent the 10^th^ and 90^th^ percentile. The error lines represent the minimal and maximal value within 1.5 quartile distances of the box. Values above 1.5 quartile distances of the box are considered outliers and are represented by a dot. Twenty independent experiments were conducted using the lower dose of belatacept (100 ng/ml) and 13 using the higher dose of belatacept (500 ng/mL). * = *p*<0.05, ** = *p*<0.01, *** = *p*<0.001, **** = *p*<0.0001.

The IC_50_ for belatacept was determined in 6 independent MLR assays with PBMCs of healthy volunteers (Fig 1). PBMCs were washed in serum-free medium and suspended in PKH67 FITC or PKH26 PE 1:50 in 1 mL Diluent C per 10 million cells (Membrane Dye Kit by Sigma-Aldrich, St. Louis, MO). After incubation of 4 minutes at room temperature, fetal bovine serum (FBS) was added to stop the incorporation of the PKH dye. Subsequently, PBMCs were washed twice in RPMI + 10% heat-inactivated FBS. Finally 5x10^4^ PKH-26 PE or PKH-67 FITC labeled (MFI>10,000) responders’ PBMCs were incubated for 1 hour with 15 different concentrations of belatacept (Bristol-Myers Squib, NYC, NY, kindly provided by the manufacturer) ranging from 0–5 mg/mL before the stimulator cells were added for 7 days. A lower concentration (100 ng/mL) and a higher concentration (500 ng/mL) for belatacept were used in further experiments.

5x10^4^ PKH-26 PE or PKH-67 FITC labeled (MFI>10,000) patients’ PBMCs, FACS-isolated T-cell memory subsets or FACS-isolated CD28^POS^ cells were incubated for 1 hour in 100 ng/mL or 500 ng/mL belatacept (Bristol-Myers Squibb, New York City, NY) or 100 or 500 ng/mL IgG (human IgG, Sigma-Aldrich, St. Louis, MO) as control. Hereafter 5x10^4^ CD3-depleted and irradiated (total dose of 40 Gy) stimulator PBMCs were added to the culture. Subsequently, the cells were incubated for 1 week at 37°C and analyzed by flow cytometry (BD FACSDiva 8.0.1, BD, Franklin Lakes, NJ).

### Flow cytometry

PBMCs were characterized (*n* = 33) before and at day 7 of the MLR. Memory subsets were defined by CCR7 and CD45RA surface expression: naïve (T_N_ cells: CCR7+CD45RA+), central-memory (T_CM_ cells: CCR7+, CD45RA-), effector-memory (T_EM_ cells: CCR7-, CD45RA-), and end-stage terminally-differentiated EMRA (T_EMRA_ cells: CCR7-CD45RA+) T-cells. At day 7 the cells were plugged with brefeldin A (Golgiplug, BD Pharm; 1 μg/mL) for 4 hours. Thereafter, the allogeneic intracellular IFNγ-production was measured. Also the intracellular IFNγ production capacity was assessed, by re-stimulating part of the cells at day 7 with phorbolmyrisate acetate (PMA) 0.05 μg/mL and ionomycin 1 μg/mL (Sigma-Aldrich, St Louis, MO). FACS- isolated memory subsets of recipient cells (*n* = 4 independent experiments) were stained before and after MLR using CD3 Brilliant Violet 510 (BioLegend), CD4 APC (BD), CD8 APC-Cy7 (BioLegend), CCR7 PE (BD) and CD45RO PE-Cy7 (BD). Intracellular staining was done using IFNγ Brilliant Violet 421 (BioLegend). The proportion of PKH67-FITC negative cells was assessed as measurement for proliferation. FACS-sorted CD28^POS^ cells (*n* = 24 independent experiments) were stained for CD28-expression and IFNγ-production after 7 days of antigen stimulation. Monoclonal antibodies used for surface marker staining and intracellular cytokine staining for PBMCs and isolated CD28^POS^ cells were CD3 Brilliant Violet 510 (BioLegend, San Diego, CA), CD4 PerCP (B DBiosciences, Frankin Lakes, NJ), CD4 APC-Cy7 (BioLegend), CD8 APC (BD), CD8 APC-Cy7 (BD Pharmingen, San Diego, CA), CCR7 PE (BD Pharmingen), CD45RA brilliant violet 421 (BioLegend), CD28 APC (BD), CD28 PerCP-Cy5 (BD) and IFNγ BV421 (BioLegend) or IFNγ APC (BD Pharmingen). The proportion of PKH-26 PE or PKH-67 FITC-negative cells was also assessed as measurement for proliferation.

### Calculating the relative inhibition by belatacept

The relative inhibition by belatacept was expressed as the proliferation of T-cells in the presence of belatacept compared to the proliferation in the presence of IgG control, which was set to 100%:

$$Relative\;inhibition=\;\frac{Proliferation\;in\;the\;presence\;of\;belatacept-Proliferation\;in\;the\;presence\;of\;IgG\;control}{Proliferation\;in\;the\;presence\;of\;IgG\;control}\;x-100\%$$

### Statistical analyses

The differences between measurements before and after 7 days of MLR, and between IgG control and belatacept, were analyzed using the Wilcoxon signed-rank test. The differences between belatacept 100 ng/mL and belatacept 500 ng/mL were analyzed using the Mann-Whitney-U test.

Graph pad prism 5.01 (GraphPad Software, San Diego, CA) was used for statistical analyses. *P*-values with a 2-sided α of 0.05 were considered statistically significant. When not otherwise specified, medians [range] are presented.

## Results

### Despite the dose-dependent inhibition by belatacept of T-cell proliferation, residual T-cell proliferation was still present when high doses of belatacept were added

Belatacept inhibited T-cell proliferation in a dose-dependent manner (Fig 1). The IC_50_ of belatacept for T-cell proliferation was 215 ng/mL [CI95% 123–376 ng/mL) in MLRs of healthy volunteers’ PBMCs. Remarkably, even at very high concentrations, belatacept could not inhibit T-cell proliferation more than ±70%, resulting in a residual proliferation of ±30%.

A concentration lower and a concentration higher than the IC50of belatacept were used in further experiments (100 ng/mL and 500 ng/mL), to ensure the inhibitory effects of belatacept were dose-dependent.

In MLRs of PBMCs of ESRD patients stimulated with allo-antigen, both CD4^POS^ and CD8^POS^ T-cells were significantly inhibited in proliferation by the lower and higher dose of belatacept, in a dose-dependent manner (Fig 1C)

### Predominance of effector-memory T-cells after allogeneic stimulation was enhanced by belatacept

**Fig 2:**
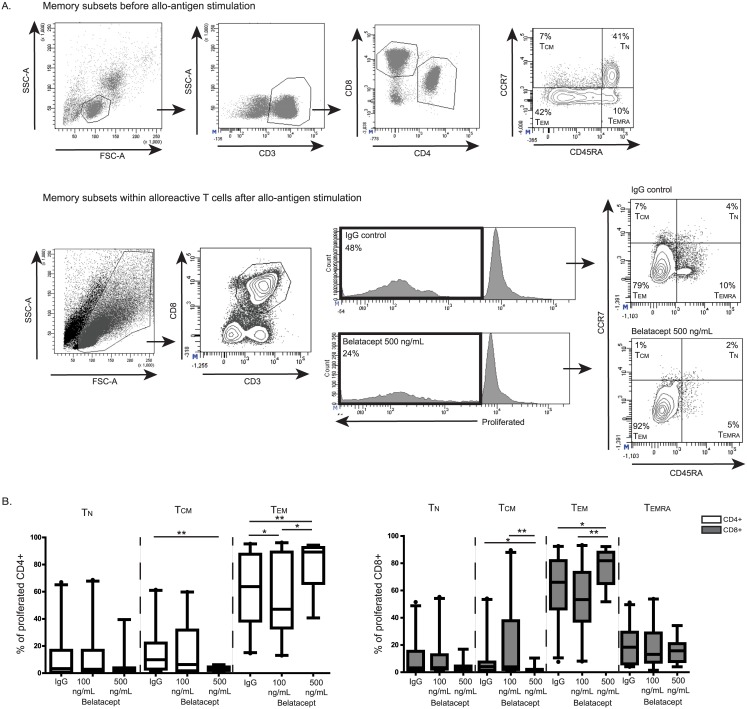
Predominance of effector-memory T-cells after allogeneic stimulation was enhanced by belatacept. A representative sample of 33 measurements is depicted for the gating strategy of the memory subsets within the proliferated, alloreactive CD8^POS^ T-cells after 7 days of allo-antigen stimulation, in the presence and abcense of belatacept, as well as the memory subsets before allo-antigen stimulation (A). Memory subsets were gated the same way for CD4^POS^ T-cells. The percentages of naïve, central memory, effector-memory and terminally-differentiated EMRA T-cells are given before allogeneic stimulation within CD4^POS^ and CD8^POS^ T cells, and after allogeneic stimulation in the presence of human IgG control, 100 ng/mL or 500 ng/mL belatacept for proliferated CD4^POS^ T-cells and for CD8^POS^ T-cells (B). N.B.: Black lines represent the medians. The upper and lower borders of the boxes represent the 10^th^ and 90^th^ percentile. The error lines represent the minimal and maximal value within 1.5 quartile distances of the box. Values above 1.5 quartile distances of the box are considered outliers and are represented by a dot. Twenty independent experiments were conducted using the lower dose of belatacept (100 ng/ml) and 13 using the higher dose of belatacept (500 ng/mL). * = *p*<0.05, ** = *p*<0.01, *** = *p*<0.001, **** = *p*<0.0001, T_N_ = naïve T-cells, T_CM_ = central-memory T-cells, T_EM_ = effector-memory T-cells, T_EMRA_ = terminally differentiated EMRA T-cells.

The T-cells that proliferated upon allogeneic stimulation were analyzed in the presence and absence of belatacept to gain more insight into the alloreactive T-cells that were less susceptible to belatacept (Fig 2A). A predominance of effector-memory T-cells was seen within the proliferated CD4^POS^ and CD8^POS^ T-cells after allo-antigen stimulation in the presence of the higher dose of belatacept (500 ng/mL). 89% [41–94%] of the alloreactive CD4^POS^ T cells expressed an effector-memory phenotype in the presence of 500 ng/mL belatacept *vs*. 64% [15–95%] for the IgG control, *p*<0.01. Similar observations were made for CD8^POS^ T cells: 82% [52–92%] in the presence of 500 ng/mL belatacept *vs*. 66% [8–93%] for the IgG control, *p*<0.01. In parallel with the proportional increase of effector-memory T cells amongst alloreactive T cells, the proportion of naïve and central-memory T cells decreased. The predominance for effector-memory T cells was not enhanced when the lower dose of belatacept was used.

### Phenotyping and function of alloreactive effector-memory T-cells

**Fig 3:**
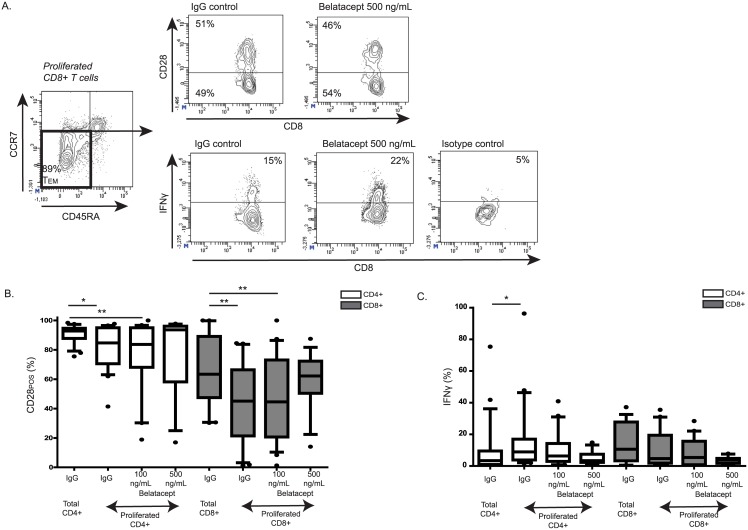
A large proportion of alloreactive T cells is CD28^POS^, and allogeneic IFNγ production is not inhibited by belatacept. A representative sample of 33 experiments is depicted for the gating strategy of CD4^POS^ and CD8^POS^ effector-memory T-cells and their CD28 expression and the intracellular IFNγ production (A). The percentage of CD28^POS^ effector-memory T-cells, (B) and the percentage of IFNγ producing cells (C) was assessed in the total and in the proliferated effector-memory CD4^POS^ and CD8^POS^ populations upon 7-day allogeneic stimulation by means of MLR. The percentages are given in the presence of human IgG control, 100 ng/mL or 500 ng/mL belatacept. N.B.: Black lines represent the medians. The upper and lower borders of the boxes represent the 10^th^ and 90^th^ percentile. The error lines represent the minimal and maximal value within 1.5 quartile distances of the box. Values above 1.5 quartile distances of the box are considered outliers and represented are by a dot. Twenty samples were used for the lower dose of belatacept (100 ng/ml) and 13 samples for the higher dose of belatacept (500 ng/mL). * = *p*<0.05, ** = *p*<0.01, *** = *p*<0.001, **** = *p*<0.0001.

Since the allo-reactive proliferated T-cells mostly consisted of effector-memory T-cells, these cells were further analyzed for CD28-expression and allogeneic IFNγ-production (Fig 3). The proliferated effector-memory T-cells were compared to the total effector-memory T-cell population (both proliferated and non-proliferated cells). Within the proliferated effector-memory CD4^POS^ T-cell population, the percentage of CD28^POS^ T-cells (85% [41–98%]) was lower than in the total CD4^POS^ effector-memory population (93% [76–98%]), *p* = 0.01, but still substantial (Fig 3B). No selection of CD28^NULL^ T-cells occurred in the presence of belatacept, since similar proportions of CD28^POS^ cells within the proliferated CD4^POS^ effector-memory T-cells were observed in the presence of the IgG control, belatacept 100 ng/mL or 500 ng/mL. In the proliferated CD8^POS^ effector-memory T-cell population 45% [1–85%] of the cells were CD28^POS^ compared to 63% [30–100%] in the total CD8^POS^ effector-memory T-cell population, *p* = 0.01 (Fig 3B). Despite adding the lower or higher dose of belatacept, similar proportions of CD28^POS^ cells were observed among the proliferated CD8^POS^ effector-memory T cells as in the IgG control (Fig 3B).

9% [2–96%] of the proliferated CD4^POS^ effector-memory T-cells and 5% [0–36%] of the proliferated CD8^POS^ effector-memory T-cells produced IFNγ upon allogeneic stimulation. The allogeneic production of this important cytokine by CD4^POS^ and CD8^POS^ effector-memory T-cells was not significantly inhibited by the lower or higher dose of belatacept. The allogeneic IFNγ production by proliferated CD4^POS^ effector-memory T-cells (9% [2–96%]) was significantly higher than the production by the total CD4^POS^ effector-memory T-cell population (3% [1–75%]), *p* = 0.03 (Fig 3C). This difference was not observed between the proliferated and total CD8^POS^ effector-memory T-cells. A fair proportion of CD4^POS^ and CD8^POS^ T cells had the capacity to produce IFNγ after re-stimulation with PMA/ionomycin (S1 Fig).

### Isolated effector-memory T cells were not inhibited in differentiation or IFNγ production by belatacept

**Fig 4:**
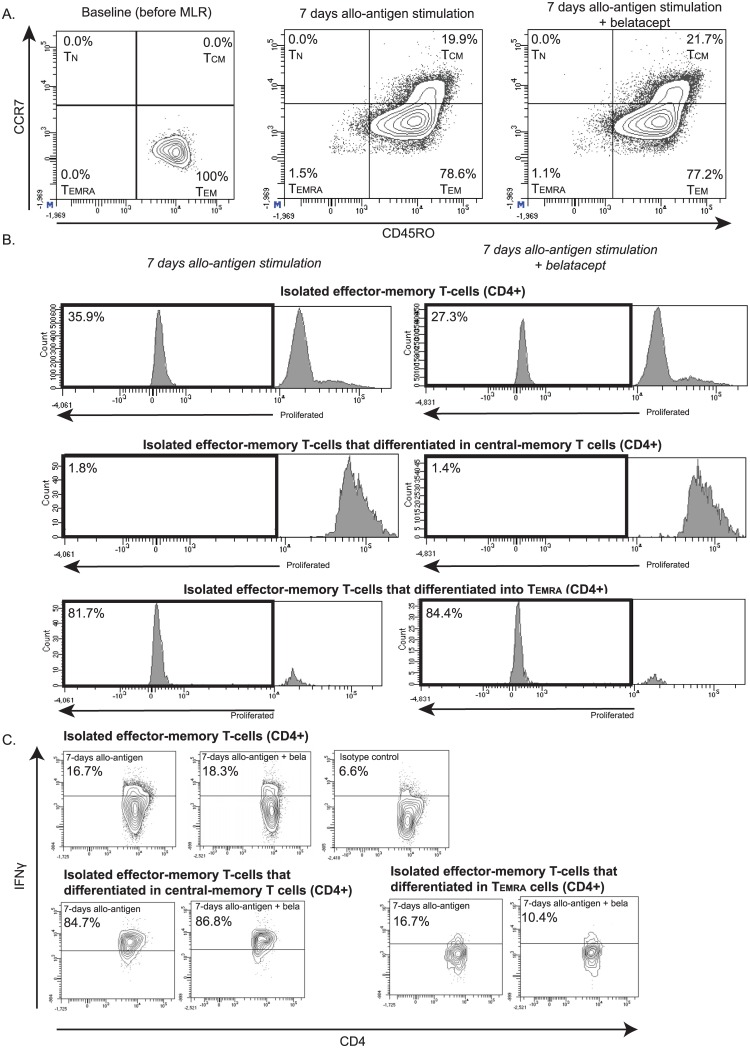
Isolated effector-memory T cells are not inhibited in differentiation or IFNγ production by belatacept. FACS-sorted effector-memory T-cells (CD3+CCR7-CD45RO+) were stimulated for 7 days with allo-antigen (purities≥99%). One of in total four independent experiments is shown in this figure. The surface expression of CCR7 and CD45RO was assessed on CD4^POS^ and CD8^POS^ T cells after 7 days of MLR to determine the differentiation of effector-memory T cells into other memory subsets; an example of CD4^POS^ isolated effector-memory T cells is shown in (A). The proliferation by the isolated effector-memory T cells, and by the cells differentiated into central-memory and end-stage terminally-differentiated effector-memory T-cells in particular, is depicted (B), as well as the allogeneic IFNγ production by these cells (C). bela = belatacept 500 ng/mL, T_N_ = naïve T-cells, T_CM_ = central-memory T-cells, T_EM_ = effector-memory T-cells, T_EMRA_ = terminally differentiated EMRA T-cells.

For detailed information regarding the experiments using isolated memory subsets, see Table B in S1 File. Sufficient cell numbers were not available for all test conditions. The differentiation of isolated effector-memory T-cells into central-memory or end-stage differentiated effector-memory T-cells was not inhibited by belatacept 500 ng/mL (Fig 4A and Table C in S1 File). The differentiation of isolated naïve or central-memory T-cells into other subsets was successfully suppressed by belatacept (S2A Fig and Table C in S1 File). Each subset, except for T_EMRA_ cells, was successfully inhibited in proliferation by 500 ng/mL belatacept (Fig 4B, S2B Fig and Table D in S1 File). The proliferation within the newly formed memory subsets, however, was high and not inhibited by belatacept, especially in the newly formed effector-memory and T_EMRA_ cells (Fig 4B and Table D in S1 File). Intracellular IFNγ concentrations were highest in the newly formed memory subsets differentiated from isolated naïve, effector-memory and T_EMRA_ cells (Table E in S1 File). Belatacept 500 ng/mL could not prevent the IFNγ production by these induced subsets (Table E in S1 File). IFNγ production by isolated effector-memory T-cells was also not blocked by belatacept (Fig 4C and Table E in S1 File). Belatacept could, however, inhibit IFNγ production by the isolated naïve and central-memory T-cells (S2C Fig), as well as in the newly formed subsets differentiated from central-memory T-cells (Table E in S1 File).

### The indirect target of belatacept, CD28, can be down-regulated by T-cells upon allogeneic stimulation, resulting in IFNγ-producing CD28^NULL^ T-cells

**Fig 5:**
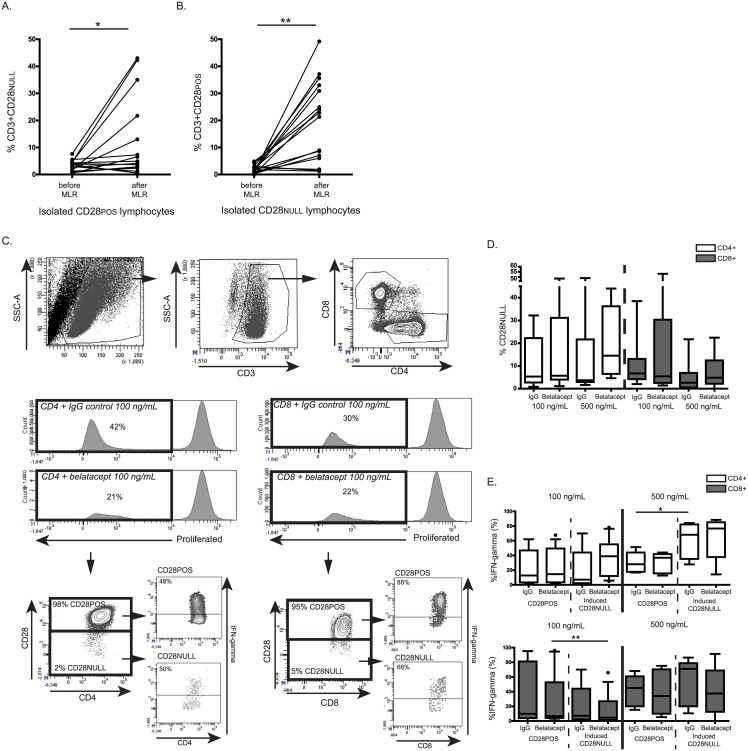
The indirect target of belatacept, CD28, can be down regulated by T-cells upon allogeneic stimulation, resulting in IFNγ-producing CD28^NULL^ T-cells. FACS-sorted CD28^POS^ T-cells (A) and FACS-sorted CD28^NULL^ T-cells (B) from n = 16 kidney-transplant candidates were stimulated for 7 days with allo-antigen (purities ≥95%). CD28-expression was assessed after 7 days of MLR and compared to the CD28-expression of the pure starting populations. Gating strategies to determine proliferated CD4^POS^ and CD8^POS^ T-cells are depicted, as well as a typical example for proliferation with and without belatacept (CD). The gating strategy for CD28 expression within proliferated CD4^POS^ and CD8^POS^ T-cells, including allogeneic IFNγ production, is depicted in a typical example (C). IFNγ expression is depicted in this typical example in the presence of 100 ng/mL belatacept. The proportion of CD4^POS^ and CD8^POS^ T-cells which lost their CD28-molecules are shown for the study population (D). The percentage of IFNγ producing cells within T-cells that remained CD28^POS^ and within T-cells that differentiated into CD28^NULL^ are shown, in the presence and absence of belatacept (E). N.B.: Black lines represent the medians. The upper and lower border of the boxes represent the 10^th^ and 90^th^ percentile. The error lines represent the minimal and maximal value within 1.5 quartile distances of the box. Values above 1.5 quartile distances of the box are considered outliers and are represented by a dot. Levels of significance in (B) were given for the difference between proliferation in the presence of belatacept and the IgG control. * = *p*<0.05, ** = *p*<0.01, *** = *p*<0.001, **** = *p*<0.0001, T_N_ = naïve T-cells, T_CM_ = central-memory T-cells, T_EM_ = effector-memory T-cells, T_EMRA_ = terminally differentiated EMRA T-cells.

To study the dynamics of CD28-expression on T-cells, pure CD28^POS^ and CD28^NULL^ cell populations were studied after allogeneic stimulation (Fig 5). A fair proportion of the isolated CD28^NULL^ T-cells up-regulated CD28, but more importantly, also a proportion of the CD28^POS^ T-cells down-regulated CD28. (Fig 5A and 5B) Therefore, CD28-expression was also studied within proliferated isolated CD28^POS^ T-cells, in the presence or absence of belatacept (Fig 5C). The inhibitory effect on isolated CD28^POS^ T-cell proliferation is depicted in S3 Fig. After allogeneic stimulation, in the presence of belatacept 100 ng/mL, 6% [1–49%] of the CD4^POS^ T-cells did not express CD28 anymore and 5% [1–53%]of the CD8^POS^ population. These percentages were similar to the percentages of CD28^NULL^ T-cells in the absence of belatacept (Fig 5D). Similar proportions of CD28^NULL^ T-cells were found for cultures with the higher concentration of belatacept.

The allogeneic IFNγ production was compared between T-cells that remained CD28^POS^ and T-cells that had down-regulated their CD28 surface molecules (Fig 5C for typical examples). In the presence of 100 ng/mL belatacept, the proportion of CD4^POS^CD28^NULL^ T-cells that produced IFNγ (39% [4–93%]) was comparable to cells that remained CD28^POS^ (15% [2–68%]), *p* = 0.08. (Fig 5E) The same was seen when 500 ng/mL belatacept was added. Within the CD8^POS^ T-cells, in the presence of 100 ng/mL belatacept, slightly more CD28^POS^ T-cells produced IFNγ than the cells that turned CD28^NULL^: 7%[0–96%] *vs*. 4% [0–66%], respectively, *p* = 0.003. This difference was not observed when 500 ng/mL belatacept was added to the cultures. Similar percentages of IFN-γ production were found for the IgG control samples, *i*.*e*. in the absence of belatacept. (Fig 5E)

## Discussion

Here, the ability of ESRD patients’ CD28^POS^ T-cells to down-regulate surface CD28 upon allogeneic stimulation was studied after belatacept was added *ex vivo*. Kidney transplantation was mimicked to explain the severe T-cell-mediated immune responses that have been observed in belatacept-treated patients.[1] Although the overall allogeneic proliferation by T-cells was inhibited by belatacept, this inhibition never reached 100% (Fig 1). Moreover, a part of the antigen-reactive T-cells down-regulated surface CD28 molecules without becoming anergic (*i*.*e*. their capacity to produce intracellular IFNy production upon allogeneic stimulation remained intact; Fig 5E). Even in the presence of belatacept, the co-stimulatory inhibitor of the CD80/86-CD28 pathway, these originally CD28^POS^ T-cellswere not susceptible to co-stimulatory blockade and are therefore a selection of the most dangerous immune cells for the allograft.[6, 7] In addition, amongst the antigen-reactive proliferated T-cells, a large proportion remained CD28^POS^ and also produced intracellular IFNγ. Explanations for the severe alloreactivity in belatacept-treated patients include the possibility that belatacept inhibits negative regulators of the immune system [15, 16]; ineffectively permeate lymph nodes and kidney tissue [17]; or the alloreactivity is the result of heterologous immunity, like EBV positive memory T cells may cross-react with donor HLA expressed on the transplanted kidney.[18] Based on our research presented here, we postulate three additional mechanisms for the severe alloreactivity in belatacept-treated patients [1]: (i) proliferation is not inhibited in all T-cells; (ii) naïve and central-memory T-cells differentiate into effector-memory T-cells, which are less susceptible to immunosuppressive drugs [19, 20]; and (iii) T-cells can down-regulate their cell surface CD28 molecule and consequently become independent of co-stimulatory signals from CD80/86.

The log [inhibitor]- *versus*—response curve of belatacept (Fig 1B) demonstrated that a plateau phase is reached for its inhibitory capacity. Even when high doses of belatacept (>1 mg/mL) were added *in vitro*, the maximum inhibition was ±70%. In the BENEFIT study, serum belatacept concentrations were not higher than 10 μg/mL [1, 21], suggesting that T-cell proliferation may also be incompletely blocked *in vivo*. The IC_50_ of belatacept found in our *in vitro* experiments (0.22 μg/mL, 95% CI 0.12–0.38 μg/mL) was similar to the serum belatacept concentrations of stable patients 2–5 years after kidney transplantation that received belatacept every 8 weeks (0.13–0.21 μg/mL).[2] Because the volume of distribution of belatacept is low [22], the concentration in lymph nodes or graft tissue is presumably even lower, which could result in even more proliferation of allo-reactive T-cells.

It is known that CD28^NULL^ T-cells are not susceptible to belatacept and can produce high amounts of effector cytokines.[5–7] When adding belatacept to patients’ PBMCs *ex vivo*, a smaller proportion of CD28^POS^ T-cells, thus a larger proportion of CD28^NULL^ T-cells was observed within the cells proliferated upon allogeneic stimulation (Figs 3B and 5D). This can be explained by a selection of CD28^NULL^ T-cells, because these cells are not susceptible to belatacept. Another explanation is that not all CD28^POS^ T-cells are inhibited by belatacept and that their CD28 co-stimulatory molecule is down-regulated, since CD28^NULL^ T-cells were present in cultures of isolated CD28^POS^ T-cells after one week of MLR. (Fig 5A and 5D) When adding the higher dose of belatacept to MLRs with patients’ PBMCs (Fig 3), the predominance of CD28^NULL^ T-cells was not observed, possibly because belatacept at this concentration sufficiently inhibited the activation of CD28^POS^ T-cells and subsequently prevented the differentiation into CD28^NULL^ T-cells. Another possibility could be that equal numbers of CD28^NULL^ T-cells upregulated CD28 as the number of CD28^POS^ T-cells that down-regulated CD28, and therefore the net-result was no increase of CD28^NULL^ T-cells. Nevertheless, apart from CD28^NULL^ T-cells, a large proportion of allo-reactive, proliferated T cells was CD28^POS^, which means that despite their surface CD28 molecules these cells were not susceptible for belatacept.

To accurately establish the dynamics of CD28-expression by alloreactive T-cells of ESRD patients in the presence of belatacept, the proportion of CD28^NULL^ T-cells was measured after one week of allogeneic stimulated pure CD28^POS^ T-cells. (Fig 5) Indeed, even in the presence of belatacept, a proportion of T-cells lost their CD28 surface molecules upon allogeneic stimulation, making them not susceptible to inhibition of the CD28-pathway. These CD28^NULL^ T-cells did not become senescent anergic, since they remained capable of producing intracellular IFNγ upon allogeneic stimulation. For CD4^POS^ and CD8^POS^ T-cells, both CD28^POS^ or newly-formed CD28^NULL^ T-cells produced comparable large amounts of allogeneic IFNγ (Fig 5E). The differentiation of CD28^POS^ T-cells into IFNγ–producing CD28^NULL^ T-cells reflects the absence of belatacept-induced anergy of these T-cells (Fig 5), possibly because of alternative routes for co-stimulation.[23]

In the present study, using PBMCs of kidney transplant candidates, the alloreactive cells mostly had effector-memory T-cell features, especially after allogeneic stimulation in the presence of belatacept (Fig 2). However, the absolute number of such responding cells were lower because belatacept inhibits T-cell proliferation (Fig 1). The predominance of effector-memory T cells is in line with previous findings from studies using animals [5] or PBMCs from healthy volunteers. [6, 7] These effector memory T-cells are less susceptible to the currently prescribed immunosuppressive drugs, like tacrolimus [19], and are especially less subject to co-stimulation blockade.[23] The predominance of effector-memory T-cells could be the result of less affected proliferation of the pre-existing effector-memory T-cells. Isolated effector-memory T cells could, however, be inhibited in proliferation by belatacept (Fig 4B and Table D in S1 File), but not in differentiation into central-memory and T_EMRA_ T cells or in IFNγ production (Fig 4B, Tables C and E in S1 File). The selection of effector-memory T-cells was evident when the higher dose of belatacept was added, since naïve and central-memory T-cells are then more sufficiently inhibited (Figs 1 and 2). In addition, the predominance of effector-memory T-cells could also be the result of differentiation of naïve and central-memory T-cells into the effector-memory phenotype upon allogeneic stimulation (S2 Fig).[24]

A limitation of our study is the low availability of patient materials, which makes it difficult to test multiple conditions, *e*.*g*. the distinction between allorecognition via the direct or indirect pathway. Also, the difference between patients and healthy controls would be an interesting question, but does not address to our initial study purpose.

In conclusion, CD28-positive, mostly effector-memory T-cells can become resistant to belatacept by down-regulating their surface CD28 molecules, indicating differentiation into highly allo-reactive CD28^NULL^ T-cells. This study provides evidence that not only CD28^NULL^ T-cells but also CD28^POS^ T-cells can mediate anti-donor responses despite belatacept treatment.

## Supporting Information

S1 Fig CD4^POS^ and CD8^POS^ T-cells from end-stage renal disease patients have a high IFNγ production capacity. The intracellular IFNγ production is depicted for both CD4^POS^ and CD8^POS^ T-cells after 7 days of allo-antigen stimulation with and without 4 hours PMA/ionomycin re-stimulation. bela = belatacept 100 ng/mL.
(EPS)

S2 Fig Belatacept inhibits differentiation, proliferation and allogeneic IFNγ production by naïve and central-memory T-cells. For detailed information about these experiments refer to Table B-E in S1 File. The differentiation of isolated T-cell memory subsets (naïve, central-memory and end-stage terminally-differentiated effector-memory T-cells) into other memory subsets is depicted in the presence and absence of 500 ng/mL belatacept (A). All starting population were ≥97% pure. The proliferation of these isolated memory subsets was assessed in the presence and absence of 500 ng/mL belatacept (B), as well as the allogeneic IFNγ production (C). bela = belatacept 500 ng/mL, T_N_ = naïve T-cells, T_CM_ = central-memory T-cells, T_EM_ = effector-memory T-cells, T_EMRA_ = terminally differentiated EMRA T-cells.
(EPS)

S3 Fig The proliferation of isolated CD28^POS^ T-cells is inhibited by belatacept. The relative inhibition of kidney-transplant candidates’ CD28^POS^-isolated CD4^POS^ and CD8^POS^ T-cells in the presence of 100 (n = 16) or 500 ng/mL (n = 8) belatacept is shown (B). The human IgG control has been set at the zero-line.
(EPS)

S1 File Table A: Patient characteristics. Table B: Detailed information about the experiments using isolated T-cell memory subsets. Table C: Differentiation by isolated T-cell memory subsets upon allo-antigen stimulation. Table D: Proliferation by isolated T-cell memory subsets upon allo-antigen stimulation. Table E: Intracellular IFNγ expression by isolated T-cell memory subsets upon allo-antigen stimulation.
(DOCX)
