# Supplementary material for: Down-Regulation of Surface CD28 under Belatacept Treatment: An Escape Mechanism for Antigen-Reactive T-Cells
Source: PLoS One. 2016 Feb 26;11(2):e0148604. doi: 10.1371/journal.pone.0148604 (PMC4769294; doi:10.1371/journal.pone.0148604)
Supplement: S1 File — Table A: Patient characteristics. Table B: Detailed information about the experiments using isolated T-cell memory subsets. Table C: Differentiation by isolated T-cell memory subsets upon allo-antigen stimulation. Table D: Proliferation by isolated T-cell memory subsets upon allo-antigen stimulation. Table E: Intracellular IFNγ expression by isolated T-cell memory subsets upon allo-antigen stimulation. (DOCX) [file pone.0148604.s004.docx]

**Supplementary Table A. Patient characteristics**

|  | **PBMC study (*n* = 33)** | **Isolated T-cell memory subsets study (*n*=4)** | **Isolated CD28^POS^ cells study (*n* = 24)** |
| --- | --- | --- | --- |
| **Age in years (median + range)** | 49 (20-75) | 70 (53-74) | 55 (19-71) |
| **Gender (female)** | 33% | 0% | 25% |
| **HLA mismatches with allo-antigen (mean ± SD)** | 4.0 (±1.3) | 3.8 (±1.5) | 4.1 (±1.4) |
| **CMV seropositive** | 70% | 50% | 71% |
| **Renal replacement therapy** | 67% | 0% | 55% |
| - **Time on renal replacement therapy in months (median + range)** | 21 (2-71) | - | 6 (1-73) |

**Table A.** CD28^POS^=CD28-positive, HLA=human leukocyte antigen, MLR=mixed lymphocyte reaction, PBMCs=peripheral blood mononuclear cells, SD=standard deviation

**Supplementary Table B. Detailed information about the experiments using isolated T-cell memory subsets**

| **No.** | **PBMCs** | **Isolated TN** | | | | **Isolated TCM** | | | | **Isolated TEM** | | | | **Isolated TEMRA** | | | |
| --- | --- | --- | --- | --- | --- | --- | --- | --- | --- | --- | --- | --- | --- | --- | --- | --- | --- |
|  | **#** | **#** | **No bela** | **bela** | **ics** | **#** | **No bela** | **bela** | **ics** | **#** | **No bela** | **bela** | **ics** | **#** | **No bela** | **bela** | **ics** |
| 1 | 39 * 10^6 | 0,07 * 10^6 | - | - | - | 0,14 * 10^6 | + | + | - | 2,7 * 10^6 | + | + | + | 0,12 * 10^6 | + | - | - |
| 2 | 51 *10^6 | 2,8 * 10^6 | + | + | + | 1,8 *10^6 | + | + | + | 1,5 * 10^6 | + | + | + | 0,3 * 10^6 | + | - | - |
| 3 | 62 * 10^6 | 1,4 * 10^6 | + | + | + | 0,7 * 10^6 | + | + | + | 2,7 * 10^6 | + | + | + | 1,6 * 10^6 | + | + | + |
| 4 | 72 * 10^6 | 0,3 * 10^6 | + | + | - | 0,12 * 10^6 | + | - | - | 0,8 * 10^6 | + | + | + | 0,05 * 10^6 | - | - | - |

**Table B.** # =number of cells; + =experiment performed, - =experiment not performed due to too low or no cell numbers; No bela =no belatacept added to culture, bela =belatacept 500 ng/mL added to culture, ics =intracellular staining for IFNγ; No. =Number of experiment performed (total n=4 independent experiments); PBMCs =Peripheral blood mononuclear cells (number of cells before FACS isolation); TCM =central-memory T cells; TEM =effector-memory T cells; TEMRA =end-stage terminally-differentiated effector-memory T cells; TN =naïve T cells

**Supplementary Table C. Differentiation by isolated T-cell memory subsets upon allo-antigen stimulation**

| **No.** | **Starting subset** | **Purity starting subset** | **Bela** | **CD4+ T cells after 7 days allo-antigen stimulation** | | | | **CD8+ T cells after 7 days allo-antigen stimulation** | | | |
| --- | --- | --- | --- | --- | --- | --- | --- | --- | --- | --- | --- |
|  |  | **% within CD3+ cells** | **ng/mL** | **TN (%)** | **TCM (%)** | **TEM (%)** | **TEMRA (%)** | **TN (%)** | **TCM (%)** | **TEM (%)** | **TEMRA (%)** |
| 1 | TN | 99,3 | 0 | - | - | - | - | - | - | - | - |
|  |  |  | 500 | - | - | - | - | - | - | - | - |
| 2 |  | 99,9 | 0 | 56,1 | 4,7 | 37,3 | 1,9 | 75,6 | 2,7 | 20,9 | 1,2 |
|  |  |  | 500 | 78,4 | 6,6 | 14,0 | 1,0 | 93,7 | 2,3 | 3,9 | 0,2 |
| 3 |  | 99,6 | 0 | 95,7 | 1,1 | 2,6 | 0,7 | 97,1 | 0,8 | 1,8 | 0,4 |
|  |  |  | 500 | 95,8 | 1 | 2,5 | 0,8 | 98,8 | 0,6 | 0,4 | 0,2 |
| 4 |  | 99,9 | 0 | 98,9 | 0,7 | 0,5 | 0 | 97,8 | 1 | 1,2 | 0,2 |
|  |  |  | 500 | 99,3 | 0,6 | 0,1 | 0,3 | 99,2 | 0,8 | 0,1 | 0 |
| 1 | TCM | 98,4 | 0 | 0,0 | 43,2 | 56,8 | 0 | 0,3 | 75,7 | 24,4 | 0 |
|  |  |  | 500 | 0,0 | 67,3 | 32,7 | 0 | 0,4 | 95,5 | 4,2 | 0 |
| 2 |  | 95,2 | 0 | 0,3 | 56,5 | 43,4 | 0,1 | 1,5 | 83,9 | 14,7 | 0,2 |
|  |  |  | 500 | 0,3 | 91 | 4 | 0,1 | 2,3 | 95,5 | 2,3 | 0,2 |
| 3 |  | 97,9 | 0 | 0,9 | 91,4 | 7,8 | 0,2 | 4,3 | 94,3 | 1,7 | 0,3 |
|  |  |  | 500 | 0,8 | 97,7 | 1,4 | 0,2 | 4,3 | 92,3 | 2,9 | 0,7 |
| 4 |  | 98,7 | 0 | 1,9 | 98,1 | 0,1 | 0 | 4 | 93,8 | 2,3 | 0 |
|  |  |  | 500 | - | - | - | - | - | - | - | - |
| 1 | TEM | 99,5 | 0 | 0 | 10,6 | 86,7 | 3,3 | 0 | 1,6 | 97 | 1,8 |
|  |  |  | 500 | 0 | 16,3 | 81,3 | 3,5 | 0 | 2,9 | 96,4 | 1,1 |
| 2 |  | 99,8 | 0 | 0,2 | 56,5 | 43,4 | 0,1 | 1,5 | 83,9 | 14,7 | 0,2 |
|  |  |  | 500 | 0,1 | 96 | 4 | 0,1 | 0,8 | 98,8 | 0,6 | 0 |
| 3 |  | 99,4 | 0 | 0 | 5,8 | 94,2 | 0,3 | 0 | 1,3 | 98,1 | 0,7 |
|  |  |  | 500 | 0 | 5,5 | 94,5 | 0,2 | 0 | 1,5 | 98,2 | 0,6 |
| 4 |  | 100 | 0 | 0 | 19,9 | 78,6 | 1,5 | 0,1 | 4,5 | 91,9 | 4 |
|  |  |  | 500 | 0 | 21,7 | 77,2 | 1,1 | 0,1 | 5,8 | 91,5 | 3,4 |
| 1 | TEMRA | 99,9 | 0 | 2,1 | 0,5 | 26,1 | 71,5 | 2 | 0,3 | 4,6 | 93,3 |
|  |  |  | 500 | - | - | - | - | - | - | - | - |
| 2 |  | 99,8 | 0 | 0,7 | 0,1 | 6,8 | 92,4 | 2,6 | 0,2 | 8,6 | 89 |
|  |  |  | 500 | - | - | - | - | - | - | - | - |
| 3 |  | 99,8 | 0 | 1,2 | 2,5 | 29,4 | 66,9 | 3,5 | 1,9 | 2 | 93 |
|  |  |  | 500 | 0,2 | 1,3 | 32 | 66,5 | 3,6 | 2,4 | 2,4 | 91,9 |
| 4 |  | 97,4 | 0 | - | - | - | - | - | - | - | - |
|  |  |  | 500 | - | - | - | - | - | - | - | - |

**Table C.** The percentage of every memory subsets is depicted within the total CD4^POS^ and CD8^POS^ T cell population after 7 days allo-stimulation.

- =experiment not conducted; bela =belatacept; No. =Number of experiment performed (total n=4 independent experiments); CM =central-memory T cells; TEM =effector-memory T cells; TEMRA =end-stage terminally-differentiated effector-memory T cells; TN =naïve T cells

**Supplementary Table D. Proliferation by isolated T-cell memory subsets upon allo-antigen stimulation**

| **No.** | **Starting subset** | **Bela** | **CD4+ T cells after 7 days allo-antigen stimulation** | | | | | **CD8+ T cells after 7 days allo-antigen stimulation** | | | | |
| --- | --- | --- | --- | --- | --- | --- | --- | --- | --- | --- | --- | --- |
|  |  | **ng/mL** | **Total (%)** | **TN (%)** | **TCM (%)** | **TEM (%)** | **TEMRA (%)** | **Total (%)** | **TN (%)** | **TCM (%)** | **TEM (%)** | **TEMRA (%)** |
| 1 | TN | 0 | - | - | - | - | - | - | - | - | - | - |
|  |  | 500 | - | - | - | - | - | - | - | - | - | - |
| 2 |  | 0 | 27,5 | 1,1 | 38,4 | 99,8 | 94,8 | 21 | 0,9 | 41,2 | 99,6 | 85,3 |
|  |  | 500 | 7 | 0,2 | 29,3 | 98,8 | 85,7 | 2,8 | 0 | 16,3 | 99,4 | 38 |
| 3 |  | 0 | 3,5 | 0,1 | 32,3 | 99 | 78,2 | 2 | 0 | 13,8 | 98,5 | 39,8 |
|  |  | 500 | 3,6 | 0,1 | 35,3 | 99,2 | 91,8 | 0,5 | 0 | 7,6 | 88,2 | 33,7 |
| 4 |  | 0 | 0,9 | 0 | 37,9 | 91,2 | 28 | 1,2 | 0 | 24,1 | 91,7 | 0 |
|  |  | 500 | 0,2 | 0 | 8 | 84,6 | 66,7 | 0 | 0 | 0 | 0 | 0 |
| 1 | TCM | 0 | 74,4 | 1,4 | 46,2 | 99,9 | *N.A.* | 31,2 | 0 | 9,5 | 99,7 | *N.A.* |
|  |  | 500 | 47,5 | 1,2 | 27 | 99,5 | *N.A.* | 6,1 | 0 | 2 | 96,4 | *N.A.* |
| 2 |  | 0 | 43,3 | 0,8 | 10,1 | 99,8 | 100 | 14,6 | 0 | 4,4 | 99,5 | *N.A.* |
|  |  | 500 | 9,7 | 0,8 | 2,2 | 99,5 | 99,2 | 1,6 | 1,9 | 0,6 | 98,6 | *N.A.* |
| 3 |  | 0 | 8,3 | 0,8 | 2,3 | 97,9 | 95 | 0,9 | 0 | 0,2 | 75 | 0 |
|  |  | 500 | 1,8 | 0,9 | 0,3 | 94,8 | 98,5 | 2,5 | 0 | 0,8 | 84,2 | *N.A.* |
| 4 |  | 0 | 0,4 | 0 | 0,3 | 54,8 | *N.A.* | 3,9 | 0 | 0,6 | 100 | *N.A.* |
|  |  | 500 | - | - | - | - | - | - | - | - | - | - |
| 1 | TEM | 0 | 35,9 | 0 | 1,8 | 37,7 | 81,7 | 3,4 | 0 | 0,4 | 3,2 | 12,4 |
|  |  | 500 | 27,3 | 0 | 1,4 | 28,6 | 84,4 | 0,9 | *N.A.* | 0 | 0,7 | 18,8 |
| 2 |  | 0 | 10,4 | 7,2 | 3,7 | 98,2 | *N.A.* | 3,3 | 0 | 0,9 | 98 | 100 |
|  |  | 500 | 7,4 | 13,4 | 3,8 | 98,3 | *N.A.* | 0,4 | 0 | 0,2 | 74 | 33,3 |
| 3 |  | 0 | 2,6 | 20 | 0,1 | 2,6 | 41,1 | 0,3 | 0 | 0 | 0,3 | 11,5 |
|  |  | 500 | 1,9 | 0 | 0,1 | 1,8 | 50,5 | 0,7 | *N.A.* | 0,6 | 0,7 | 17,1 |
| 4 |  | 0 | 4,1 | 0 | 2,6 | 4,1 | 32,2 | 0,3 | *N.A.* | 1,2 | 0,3 | 1,3 |
|  |  | 500 | 3,1 | *N.A.* | 1,4 | 3,3 | 25,6 | 0,3 | 0 | 0 | 0,2 | 4,7 |
| 1 | TEMRA | 0 | 22,2 | 0 |  | 87 | 3,6 | 3 | 0 | *N.A.* | 55,9 | 0,2 |
|  |  | 500 | - | - | - | - | - | - | - | - | - | - |
| 2 |  | 0 | 22,4 | 0 | *N.A.* | 94,2 | 1,5 | 7,9 | 0 | *N.A.* | 87,5 | 0,5 |
|  |  | 500 | - | - | - | - | - | - | - | - | - | - |
| 3 |  | 0 | 34,6 | 0 | 0 | 79,1 | 10,1 | 0,4 | 0 | 0 | 23,4 | 0,2 |
|  |  | 500 | 36,4 | 0 | 0 | 79,6 | 10,8 | 0,6 | 0 | 0 | 31,4 | 0,2 |
| 4 |  | 0 | - | - | - | - | - | - | - | - | - | - |
|  |  | 500 | - | - | - | - | - | - | - | - | - | - |

**Table D.** The percentage of proliferation is given within the total CD4^POS^ and CD8^POS^ T cell population, and within the CD4^POS^ and CD8^POS^ memory subsets after 7 days allo-stimulation.

-=experiment not conducted; bela= belatacept; No.=Number of experiment performed (total n=4 independent experiments); N.A.=not applicable (not enough cells to assess this measurement); TCM=central-memory T cells; TEM=effector-memory T cells; TEMRA=end-stage terminally-differentiated effector-memory T cells; TN=naïve T cells

**Supplementary Table E. Intracellular IFNγ expression by isolated T-cell memory subsets upon allo-antigen stimulation**

| **No.** | **Starting subset** | **Bela** | **CD4+ T cells after 7 days allo-antigen stimulation** | | | | | **CD8+ T cells after 7 days allo-antigen stimulation** | | | | |
| --- | --- | --- | --- | --- | --- | --- | --- | --- | --- | --- | --- | --- |
|  |  | **ng/mL** | **Total (%)** | **TN (%)** | **TCM (%)** | **TEM (%)** | **TEMRA (%)** | **Total (%)** | **TN (%)** | **TCM (%)** | **TEM (%)** | **TEMRA (%)** |
| 1 | TN | 0 | - | - | - | - | - | - | - | - | - | - |
|  |  | 500 | - | - | - | - | - | - | - | - | - | - |
| 2 |  | 0 | 37,7 | 15,8 | 83,3 | 24,8 | 14,7 | 27,1 | 16,7 | 94,1 | 43 | 8,8 |
|  |  | 500 | 16,4 | 16,7 | 94,5 | 32,3 | 8,3 | 12,7 | 22,9 | 93,9 | 51,8 | 22,3 |
| 3 |  | 0 | 2,7 | 2,9 | 74,3 | 5,4 | 9,7 | 3,4 | 3,9 | 84,8 | 13,7 | 18,1 |
|  |  | 500 | 2,5 | 2,7 | 63,8 | 5,1 | 6,3 | 2,7 | 4,1 | 64,4 | 49 | 12 |
| 4 |  | 0 | - | - | - | - | - | - | - | - | - | - |
|  |  | 500 | - | - | - | - | - | - | - | - | - | - |
| 1 | TCM | 0 | - | - | - | - | - | - | - | - | - | - |
|  |  | 500 | - | - | - | - | - | - | - | - | - | - |
| 2 |  | 0 | 16,6 | *N.A.* | 12,1 | 12,3 | 12,3 | 13,8 | 11,6 | 10,3 | 36,9 | 16,7 |
|  |  | 500 | 11 | *N.A.* | 8,5 | 14 | 4,2 | 8,3 | 11,1 | 7,7 | 21,1 | 25 |
| 3 |  | 0 | 8,2 | *N.A.* | 7,2 | 6,4 | 1,7 | 5,2 | 3,2 | 7 | 0 | 0 |
|  |  | 500 | 4,1 | *N.A.* | 4,1 | 1,5 | 0,7 | 2,6 | 2,4 | 2,7 | 5,3 | 0 |
| 4 |  | 0 | - | - | - | - | - | - | - | - | - | - |
|  |  | 500 | - | - | - | - | - | - | - | - | - | - |
| 1 | TEM | 0 | 16,7 | *N.A.* | 84,7 | 10 | 10,7 | 4,9 | *N.A.* | 76,2 | 2 | 6,6 |
|  |  | 500 | 18,3 | *N.A.* | 86,8 | 10,4 | 10,4 | 5,9 | *N.A.* | 79,9 | 2,2 | 8,7 |
| 2 |  | 0 | 9,6 | *N.A.* | 8 | 5,3 | 8,3 | 5,6 | *N.A.* | 5,9 | 25,3 | 0 |
|  |  | 500 | 10 | *N.A.* | 8,8 | 2,3 | 5,5 | 4,1 | *N.A.* | 6 | 4 | 0 |
| 3 |  | 0 | 3,3 | *N.A.* | 57,2 | 2,2 | 2,9 | 2 | *N.A.* | 60,3 | 1,2 | 0 |
|  |  | 500 | 3,7 | *N.A.* | 58,3 | 2,7 | 3,5 | 3,1 | *N.A.* | 59,9 | 2,1 | 1,4 |
| 4 |  | 0 | 20,9 | *N.A.* | 82,3 | 3,8 | 1,7 | 2,3 | *N.A.* | 53,6 | 1 | 1,3 |
|  |  | 500 | 24,3 | *N.A.* | 85,5 | 4,3 | 1,5 | 2,8 | *N.A.* | 59 | 1,1 | 1,6 |
| 1 | TEMRA | 0 | - | - | - | - | - | - | - | - | - | - |
|  |  | 500 | - | - | - | - | - | - | - | - | - | - |
| 2 |  | 0 | - | - | - | - | - | - | - | - | - | - |
|  |  | 500 | - | - | - | - | - | - | - | - | - | - |
| 3 |  | 0 | 17,4 | *N.A.* | *N.A.* | 26,5 | 16,2 | 4,6 | *N.A.* | *N.A.* | 21,9 | 0,9 |
|  |  | 500 | 20,1 | *N.A.* | *N.A.* | 28,4 | 19,3 | 5,9 | *N.A.* | *N.A.* | 20,6 | 1,3 |
| 4 |  | 0 | - | - | - | - | - | - | - | - | - | - |
|  |  | 500 | - | - | - | - | - | - | - | - | - | - |

**Table E.** The percentage of IFNγ production is given within the total CD4^POS^ and CD8^POS^ T cell population, and within the CD4^POS^ and CD8^POS^ memory subsets after 7 days allo-stimulation.

-=experiment not conducted; bela= belatacept; No.=Number of experiment performed (total n=4 independent experiments); N.A.=not applicable (not enough cells to assess this measurement); TCM=central-memory T cells; TEM=effector-memory T cells; TEMRA=end-stage terminally-differentiated effector-memory T cells; TN=naïve T cells
